# Supplementary material for: Responses to Medical Treatment in 192 Patients with Pancreatic Neuroendocrine Neoplasms Referred to the Copenhagen Neuroendocrine Tumour Centre in 2000–2020
Source: Cancers (Basel). 2024 Mar 18;16(6):1190. doi: 10.3390/cancers16061190 (PMC10968806; doi:10.3390/cancers16061190)
Supplement: Supplementary file 1 [file cancers-16-01190-s001.zip › cancers-2888957-supplementary.pdf]

SUPPLEMENTARY

Figure S1

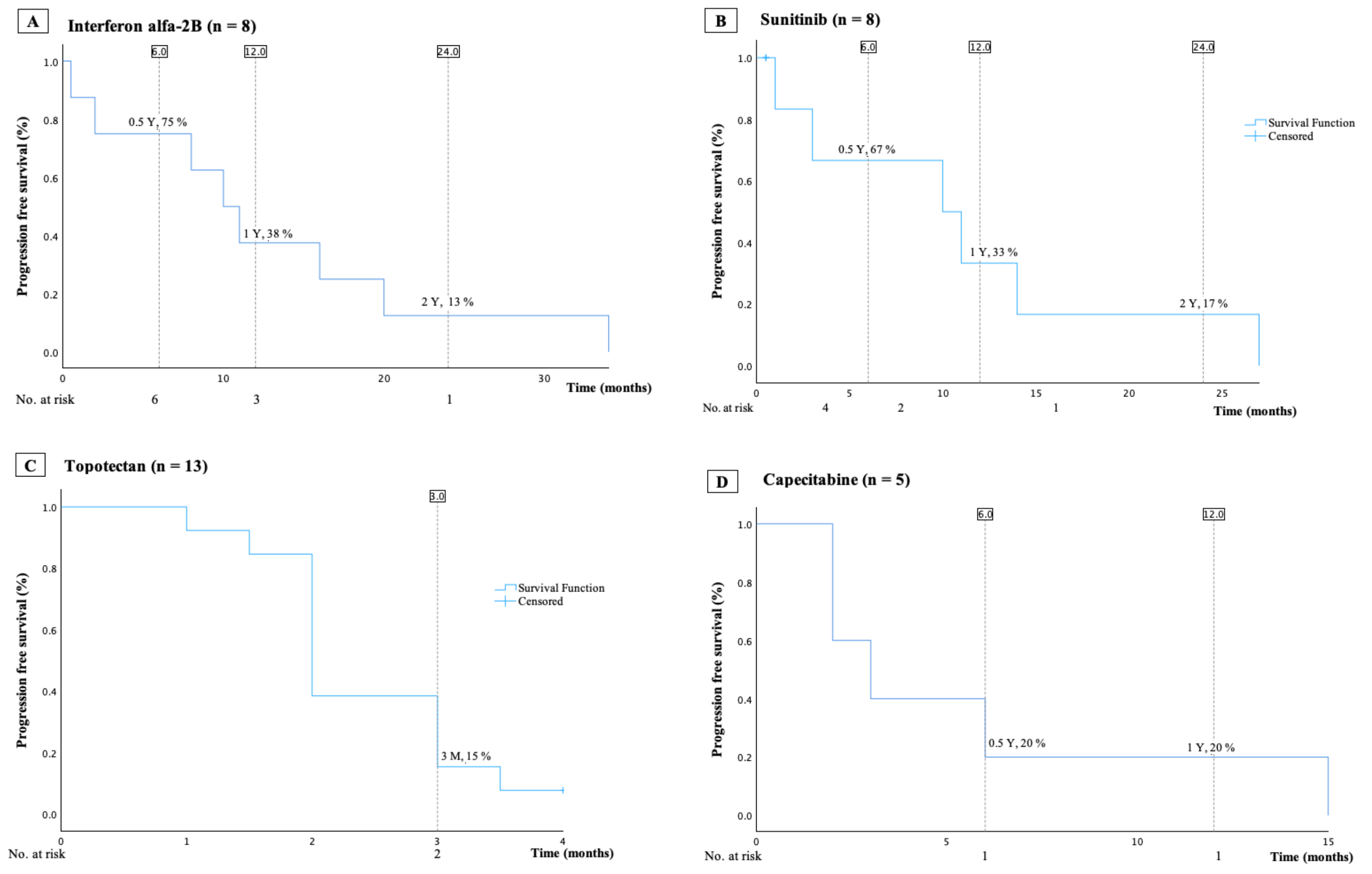

Figure S1. Kaplan-Meier curves presenting overall progression free survival in patients treated with interferon alfa-2b (mean age was 61 (range: 38-80) years, median Ki-67 was 10 (range: 2-20) %, median PFS was 10 (95%-CI: 5.8-14.2) months, A), sunitinib (mean age was 56 (range: 38-74) years, median Ki-67 was 10 (range: 3-30) %, median PFS was 10 (95%-CI: 0.4-19) months, B), topotecan (mean age was 59 (range: 33-76) years, median Ki-67 was 75 (range: 5-100) %, median PFS was 2 (95%-CI: 1.7-2.3) months, C) and capecitabine (mean age 55 (range: 38-75) years, median Ki-67 was 17 (range: 5-90)%, median PFS was 2 (95%-CI: 0.9-5.2) months, D). Survival data was missing for one patient treated with interferon alfa-2b.
